# Is it time to switch to a formulation other than the live attenuated poliovirus vaccine to prevent poliomyelitis?

**DOI:** 10.3389/fpubh.2023.1284337

**Published:** 2024-01-08

**Authors:** Christian Albert Devaux, Pierre Pontarotti, Anthony Levasseur, Philippe Colson, Didier Raoult

**Affiliations:** ^1^Laboratory Microbes Evolution Phylogeny and Infection (MEPHI), Aix-Marseille Université, IRD, APHM, Institut Hospitalo-Universitaire Méditerranée Infection, Marseille, France; ^2^Centre National de la Recherche Scientifique (CNRS-SNC5039), Marseille, France

**Keywords:** poliovirus, live attenuated virus vaccine, revertant virus, recombinant virus, nonhuman primate

## Abstract

The polioviruses (PVs) are mainly transmitted by direct contact with an infected person through the fecal-oral route and respiratory secretions (or more rarely via contaminated water or food) and have a primary tropism for the gut. After their replication in the gut, in rare cases (far less than 1% of the infected individuals), PVs can spread to the central nervous system leading to flaccid paralysis, which can result in respiratory paralysis and death. By the middle of the 20th century, every year the wild polioviruses (WPVs) are supposed to have killed or paralyzed over half a million people. The introduction of the oral poliovirus vaccines (OPVs) through mass vaccination campaigns (combined with better application of hygiene measures), was a success story which enabled the World Health Organization (WHO) to set the global eradication of poliomyelitis as an objective. However this strategy of viral eradication has its limits as the majority of poliomyelitis cases today arise in individuals infected with circulating vaccine-derived polioviruses (cVDPVs) which regain pathogenicity following reversion or recombination. In recent years (between January 2018 and May 2023), the WHO recorded 8.8 times more cases of polio which were linked to the attenuated OPV vaccines (3,442 polio cases after reversion or recombination events) than cases linked to a WPV (390 cases). Recent knowledge of the evolution of RNA viruses and the exchange of genetic material among biological entities of the intestinal microbiota, call for a reassessment of the polio eradication vaccine strategies.

## 1 Introduction

Although there have been no cases of serotype 2 wild poliovirus for more than 20 years, vaccine continued for years with the oral poliovirus vaccine (OPV2) as part of trivalent vaccine (tOPV, containing serotype 1, 2, and 3). However, OPV2 was reported to generate paralytic serotype 2 vaccine-derived poliovirus (cVDPV2) outbreaks in several continents even after withdrawal of OPV2 in April 2016 (1). It represents an obstacle to achieving polio eradication and populations with low immunization coverage are particularly at risk of cVDPV2 spread (as well as cVDPV1 and cVDPV3).

More recently, outbreaks of cVDPV2 have been increasing in frequency leading Christian Bréchot, President of the Global Virus Network, and his colleagues Chumakov et al. (2) to publish a perspective paper in the New England Journal of Medicine entitled “Choosing the right path toward polio eradication”, in which they make the following observation: “*The Global Polio Eradication Initiative (GPEI), launched 34 years ago, aimed to eradicate poliomyelitis by 2000. The chosen strategy was to stop circulation of wild polioviruses, following the successful example of smallpox eradication. The task, however, turned out to be much more challenging than eradicating smallpox had been, since there are hundreds of asymptomatic poliovirus infections for each paralytic case that occurs, which substantially complicates critical surveillance. Aside from challenges inherent in vaccine delivery in some countries, another reason for the failure to eradicate polio were outbreaks caused by cVDPV strains that emerged from viruses used in OPV. Thus, to actually eradicate poliovirus, the use of OPV must also be stopped*” and they suggest that “*the approach used by the polio-eradication campaign needs reevaluation*.” A commentary on this publication was posted by John et al. (3) who wrote: “*we agree that the use of oral polio vaccine (OPV) must also be stopped to eradicate polio. Eliminating wild poliovirus circulation with OPV and eliminating vaccine-virus polio with the use of inactivated polio vaccine (IPV) is a time-tested model*.”

What can explain these recent doubts about the effectiveness of the polio vaccine strategy? Should we speak of a failure of the global polio eradication strategy? Do we want to eliminate polioviruses or poliomyelitis? Is relying solely on outbreak response better than using OPV for preventive immunization? How to fill the gaps in mucosal immunity and stop silent virus circulation if we choose to replace OPVs with IPVs? The answers are likely different depending on whether we address countries in which polio has been eradicated or countries that need OPV to control polioviruses transmission.

## 2 Poliovirus, a virus with gastrointestinal tropism which can cause flaccid paralysis typical of poliomyelitis

**Figure 1:**
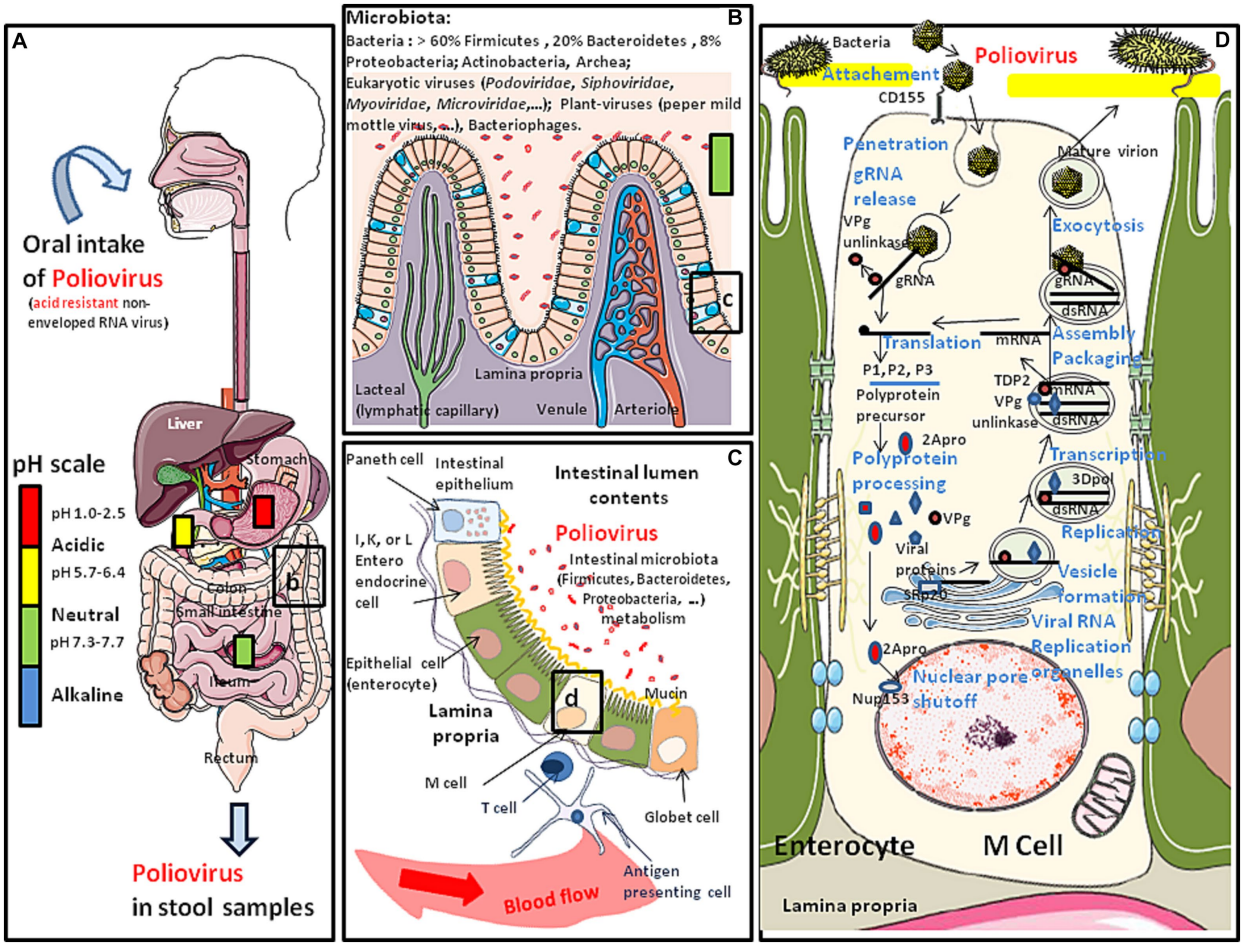
Schematic representation of the oral transmission of polioviruses (contained in water or food) and their colonization of the gastrointestinal tract. Major risk factors for poliovirus transmission include poor sanitation and hygiene conditions, high population density and tropical/subtropical climate. **(A)** Polioviruses infect humans by the fecal-oral route. This acid resistant virus can travel through the stomach (pH: 1–2) and reach the intestine. **(B)** Once in the intestine (pH: 7.3–7.7), the poliovirus (WPV or OPV) meets the commensal microbiota and the first antiviral defense such as the mucus layer, protective peptides, immune cells expressing CD103 and KLRG1 and is able to bind the E-cadherin found at the surface of epithelial cells (for details see (4, 5). However, the interaction of poliovirus capsid with lipopolysaccharide of bacteria enhances virion stability and receptor attachment (6, 7). **(C)** The polioviruses which manage to pass the first barrier of defense encounter a second barrier which is the intestinal epithelium, protecting the host against intruder transmigration. On this intestinal epithelium, polioviruses found their CD155 receptor on M cells. **(D)** Simplified model of replication cycle of polioviruses (see the main text of this paper for more details).

Polioviruses are small acid resistant, non-enveloped single positive-stranded RNA viruses with an icosahedral capsid. There are three immunologically distinct serotypes (1, 2, and 3) of wild polioviruses (WPVs), originally defined by their patterns of reactivity with neutralizing antibodies. Polioviruses are transmitted through the fecal-oral or respiratory routes. In the large majority of cases, the consequence of exposure to the virus is a transient viremia associated with poliovirus intestinal replication (Figure 1). Infection can be confined to the gut by antibodies maternally acquired or induced either by a previous infection or vaccine. However, polioviruses can also pass into the bloodstream and can ultimately infect the motor neurons of the spinal cord, inducing serious damage to the central nervous system (CNS). Polioviruses access the CNS by either crossing the blood-brain barrier (8), or through retrograde axonal transport in peripheral nerves (9, 10) The clinical consequence of viruses spreading into the CNS is flaccid paralysis which is typical of poliomyelitis. This neuropathologic evolution concerns far less than 1% of subjects infected with a WPV, but can lead to respiratory paralysis and death. Depending upon the sites of WPV replication into the CNS, it may affect either skeletal muscles (spinal poliomyelitis), respiratory muscles (bulbar poliomyelitis), or both (bulbo-spinal poliomyelitis).

In 1955, Jonas Salk licensed an inactivated polio vaccine (IPV). Although IPV protected vaccinated children against WPV-induced poliomyelitis, it did not prevent the human-to-human transmission of the poliovirus. Another type of polio vaccine, a mixture of three live attenuated strains for each of the three serotypes of PVs, known as the oral polio vaccine (OPV), was developed by Sabin (11). The formulation of the attenuated OPV, which was easy to administrate, efficient at protecting vaccinated children against poliomyelitis, and efficient at triggering mucosal immunity capable to block human-to-human fecal-oral transmission of PVs, was considered to be the ideal candidate for mass vaccination campaigns and it has proven its effectiveness. However, the risk with the use of attenuated vaccines as part of a massive vaccination strategy is that of reversion to the pathogenic phenotype. Loss of OPV attenuation can result from genetic recombinations due to the presence of a multitude of viruses which coexist at the intestinal level. Although the commensal intestinal microbiota is usually considered to be a barrier to pathogenic agents, there is evidence that the intestinal microbiota can contribute to increase poliovirus infection, replication, recombination and transmission. It has been reported (6), that by depleting the intestinal microbiota of mice with antibiotic treatment prior to inoculation with poliovirus, the antibiotic-treated mice become less susceptible to poliovirus-induced disease and viral replication in their intestine is reduced. Exposure to bacteria or their surface polysaccharides, including lipopolysaccharide (LPS) and peptidoglycan, was also reported to enhance poliovirus infectivity. Of the purified bacterial components tested, LPS was the most potent enhancer of poliovirus infectivity. The molecular basis of this cooperation between bacteria and enteroviruses remains to be further explored. It has been found that polioviruses bind to the bacterial LPS which enhances virion stability and cell attachment by increasing binding to the viral receptor (7). Robinson et al. (7) also found that a VP1-T99K mutation reduces the attachment of polioviruses to bacterial LPS and that the ratio between VP1-T99K variant virus and wild type viruses in feces is reduced. It is also possible that LPS stimulates the overexpression of the viral receptor at the cell surface. Besides, it was reported that some bacterial strains increased co-infection by different polioviruses, promoting genetic recombination between different viruses, which may enable them getting rid of deleterious mutations and restoring their viral fitness (12).

At a time when knowledge is rapidly developing on the intestinal microbiota and the multitude of viruses that use intestinal cells for their replication, is it still reasonable to use live attenuated polioviruses for worldwide poliomyelitis vaccination? This question is all the more pressing as there is currently a very unfavorable imbalance between the number of poliomyelitis cases due to natural infections by a poliovirus (WPVs) and cases linked to viruses reverting from the attenuated poliovirus vaccine preparation (cVDPVs) or the emergence of recombinant viruses. In addition, recombination between polioviruses and other enteroviruses has been also documented and represent a source of concern.

## 3 Replication cycle of polioviruses

The poliovirus icosahedral particle consists of 60 copies of each the capsid proteins VP1, VP2, VP3, and VP4 (13). The three largest proteins (VP1 to VP3) form the outer surface, while VP4 is an internal capsid protein. The host range and tissue tropism of these viruses are determined by capsid proteins (14). These viruses bind to the domain 1 of the extracellular immunoglobulin-like portion of the CD155 molecule to enter intestinal villous microfold cells (M cells), where they replicates (15). M cells are specialized epithelial cells of the gut-associated lymphoid tissues (GALT) that deliver luminal antigens to the underlying immune system after being transported to the basolateral membrane of M cells (16). Attachment to CD155 triggers endocytosis of the poliovirus particles inducing a conformational change in the capsid structure, externalizing the VP4. The hydrophobic NH2-terminal segment of VP4 inserts into the phospholipid sheets of the endosome membrane and creates a pore through which the viral genome can be released into the cytoplasm. A complete 7,410 nucleotides sequence of the poliovirus type 1 genome has been obtained from cloned cDNA (17, 18). From its 5′-end to the 3′-end, the genome first contain a 5′-end non-coding region of 743 nucleotides (nt) organized in structural stem-loop domains that precede an initiation codon. This region plays an important role in RNA synthesis and the initiation of translation. The coding region (the open reading frame starts 743 bases from the 5′-end of the mRNA and extends to a termination codon 71 bases from the 3′-end), contains the segments P1 (the 1A, 1B, 1C, and 1D subsegments encoding the VP4, VP2, VP3 and VP1 capsid components), P2 (2A, 2B, 2C) and P3 (3A, 3B, 3C, and 3D), respectively. The internal ribosome entry site (IRES) located within the 5′-end of the PV genome recruits cellular initiation factors which contribute to initiate RNA translation by the ribosome (the IRES allows the translation in conditions of the cleavage, and therefore inactivation, of a component of a cap-binding translation initiation complex elF4G). Translation leads to the synthesis of a single polyprotein precursor containing VP4, VP2, VP3 and VP1, P2, and P3. The 2A^pro^ protease contributes to the shutoff of cellular gene expression and hijacks the cellular machinery of protein synthesis for the benefit of the virus. The polyprotein precursor is then processed by virus-encoded proteinases (2A^pro^, 3C^pro^, and 3CD^pro^), to produce the P1, P2 and P3 precursors as well as the nonstructural proteins (19). During translation the first proteinase, 2A^pro^, cleaves the P1 precursor containing the amino acid sequences of the four mature capsid proteins (VP4, VP2, VP3, and VP1). Cleavage of P1 occurs soon after the ribosomes went through the 2A^pro^ coding sequences and produced active enzyme (20). Proteases 2A^pro^ and 3C^pro^ process the precursor proteins into mature proteins. Protease 3CD^pro^ processes P1 into VP0, VP3 and VP1. The cleavage of VP0 into VP4 and VP2 is believed to be autocatalytical, triggered by conformational changes upon assembled virion maturation. The 2A^pro^ also cleaves nuclear pore complex components such as Nup153 and p62, resulting in a cytoplasmic accumulation of cellular nuclear proteins such as SRp20 (a splicing factor) which acts as an important IRES trans-acting factor for poliovirus translation (21). The P3 major cleavage produces 3AB and CD then the minor cleavage produces 3A, VPg, 3C and 3D^pol^. The 3′-end of the genome consists of a short non-coding region of about 34 nucleotides which is also implicated in RNA replication (see Figure 1D). Translation of the genomic RNA also produces the RNA-dependent RNA polymerase (replicase) 3D^pol^. A molecular complex including the 3D^pol^ and 3CD subunits allow post-transcriptional modification of the VPg genome-linked peptide (or 3B, a 22 amino acid small protein bound to the 5′-terminal uridylic acid of the virus RNA) which is in turn used as a primer to replicate the genomic RNA, starting from the polyA tail. This takes place within phosphatidylinositol-4-phosphate (PI4) lipid enriched replication vesicles after remodeling of intracellular membranes (which involves Arf1 GTPase, PI4KIIIβ kinase, and GBF1 guanine nucleotide exchange factor) to generate a specialized site for RNA replication (22, 23). First a minus strand is synthesized and used as template to produce a lot of positive strand RNAs to join the cytopasmic pool of messenger RNAs or to be encapsidated to form new virions. A cellular nuclear protein redistributed in the cytoplasm as a result of 2A^pro^-dependent nuclear pore shut-off is the 5′-tyrosyl-DNA phosphodiesterase-2 (TDP2), a DNA repair enzyme identified as the source of VPg unlinkase activity that cleaves the protein-RNA covalent linkage of VPg at the 5′ end of the newly-synthesized positive RNAs that enter the translation pool (24, 25). This cleavage may be essential to control the balance between the translation, replication, and packaging functions of viral RNA. This round of replication can also create a double stranded RNA (dsRNA) (26). The virions assemble at the membranous replication vesicles then bud and are released at the cell membrane which is frequently associated with cell lysis or are released by exocytosis (21, 27, 28).

## 4 The fight against polio since the middle of the20th century

A major outbreak of WPV (over 27,000 cases) occurred in New York City in 1916 with over 2,000 deaths in the NY City and more than 6,000 deaths in the United States (https://www.sciencemuseum.org.uk/objects-and-stories/medicine/polio-20th-century-epidemic; accessed on August 28, 2023). Another US outbreak occurred in 1952 killed over 3,000 people. By the middle of the 20th century, the wild poliovirus (WPV1, WPV2 and WPV3) had spread worldwide and are supposed to have killed or paralyzed over half a million people every year. In 1955, Jonas Salk licensed an inactivated polio vaccine (IPV) and after 6 years of using this vaccine, the annual number of polio cases in the US dropped by more than 99%. Although IPV protected vaccinated children against the most severe effects of poliovirus infection, it did not prevent the human-to-human transmission of the virus. Another type of poliovirus vaccine, the oral polio vaccine (OPV), was developed by Sabin (11). The formulation, easy to administrate and capable to block human-to-human transmission of poliovirus, consists of a mixture of three live attenuated strains for each of the three serotypes of PVs. OPV was considered to be the ideal candidate for mass vaccination campaigns. Since 1988, OPV vaccine have done the heavy work towards the global eradication of poliomyelitis, an objective known as the Global Poliomyelitis Eradication Initiative (GPEI) which was adopted at the 41th World Health Assembly. Since then, poliomyelitis was effectively eradicated from most countries (29).

**Figure 2:**
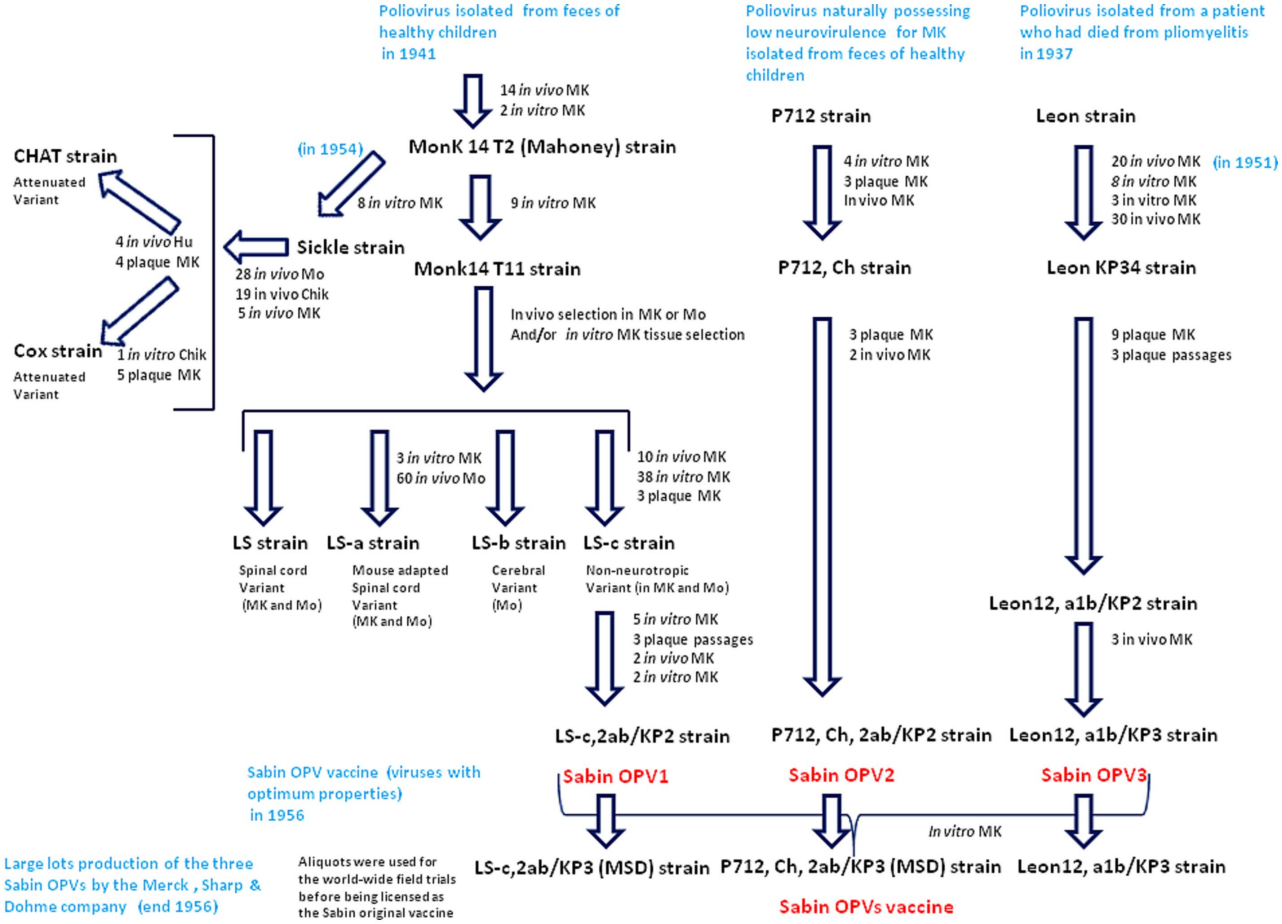
Passage histories from the wild type polioviruses to isolate the Sabin OPV1, OPV2 and OPV3. The viruses were replicated either *in vivo* on monkey (MK) or mouse (Mo) or *in vitro*. The *in vitro* cultures used cells from different tissues (cynomolgus monkey kidney cells, rhesus monkey testicular or skin tissues). At the different stages of the selection, the strains where tested for neurovirulence in cynomolgus monkeys inoculated intraspinally and those possessing the least residual neurovirulence, were selected. Culture on chik (Chik) embryos, were also used for other strains (CHAT and Cox) replicated *in vivo* in human (Hu) and was used for selection of the CHAT strain. Large batches of the three type of Sabin OPVs were produced by the MSD company using cultures of monkey kidney cells transformed by the SV40 susceptible to poliovirus infection and replication.

There are only very slight genetic differences between the wild type polioviruses responsible for human paralysis (WPV1, WPV2 and WPV3) and the three attenuated strains (Sabin OPV1, OPV2 and OPV3) that entered into the formulation of the oral anti-poliovirus vaccine (30, 31). The OPV1 (LS-c, 2ab/KP_2_ of 10/10/56) originally derives from the Mahoney virus isolated in 1941. It was first replicated *in vivo* in monkeys and then cultured *in vitro* on monkey testicular tissue to obtain the Monk14 T11 virus, from which four separate viral lines were obtained (30). The four variants were designated LS (spinal cord variant), LS-a (spinal cord variant), LS-b (cerebral variant), and LS-c (non-neurotropic variant). The progeny viruses were tested for neurovirulence in cynomolgus monkeys inoculated intraspinally. The LS-c, 2ab strain was selected because it possessed the least neurovirulence and was used for the formulation of Sabin OPV1 (30). The complete sequence of the Sabin 1 strain genome was reported in 1982 (32). The genome of Sabin OPV1 differs from that of the WPV1 Mahoney strain by 57 point mutations, 21 of which are nonsynonymous. Notably, most WPVs do not proliferate in mice and 54 nucleotide changes, leading to 20 amino acid substitutions in the virus polyprotein, were found in the comparison between the WPV1 (Mahoney virus) and the spinal cord variant LS-a strain, underlying mouse neurovirulence (18). Other live attenuated strains (CHAT and Cox) originating from the Mahoney virus were isolated after passages in monkey, mouse, and chicken embryo cells (33, 34). CHAT, which had shown favorable characteristics in terms of its restricted capacity to propagate from vaccinees, was used to immunize millions of individuals in several countries (35). The CHAT vaccine was used in a monovalent form, together with monovalent versions of Sabin OPV2 and Sabin OPV3. The OPV2 (P712, Ch, 2ab/KP_2_ of 10/10/56) was derived from the P712 virus, a naturally occurring strain of WPV2 possessing low neurovirulence. It was cultured four times in monkey kidney cell cultures and submitted to three consecutive passages in culture plates before being replicated in chimpanzees (P712, Ch), and further purified by three consecutive passages from single plaques before being selected as the vaccine virus. After two additional passages in cynomolgus monkey kidney cell cultures, it was used to prepare the formulation of Sabin OPV2. The OPV3 (Leon 12a1b/KPs of l0/10/56), was derived from the Leon virus obtained from the brain and spinal cord of a 11 years-old child who had died from poliomyelitis in the USA in 1937. This virus was replicated in monkeys through administration by the intracerebral route for 20 passages, followed by its replication in monkey testicular tissue culture and kidney cells from which the Leon KP34 was isolated. The progeny of nine selected plaques was subjected to neurovirulence evaluation. The less neurovirulent strain was used to prepare the formulation of Sabin OPV3. Large batches of the three types of Sabin OPVs were mainly produced by the Merck, Sharp & Dohme (MSD) in cultures of monkey kidney cells transformed by the SV40 virus but, other manufacturers received the Sabin virus (Figure 2).

**Figure 3:**
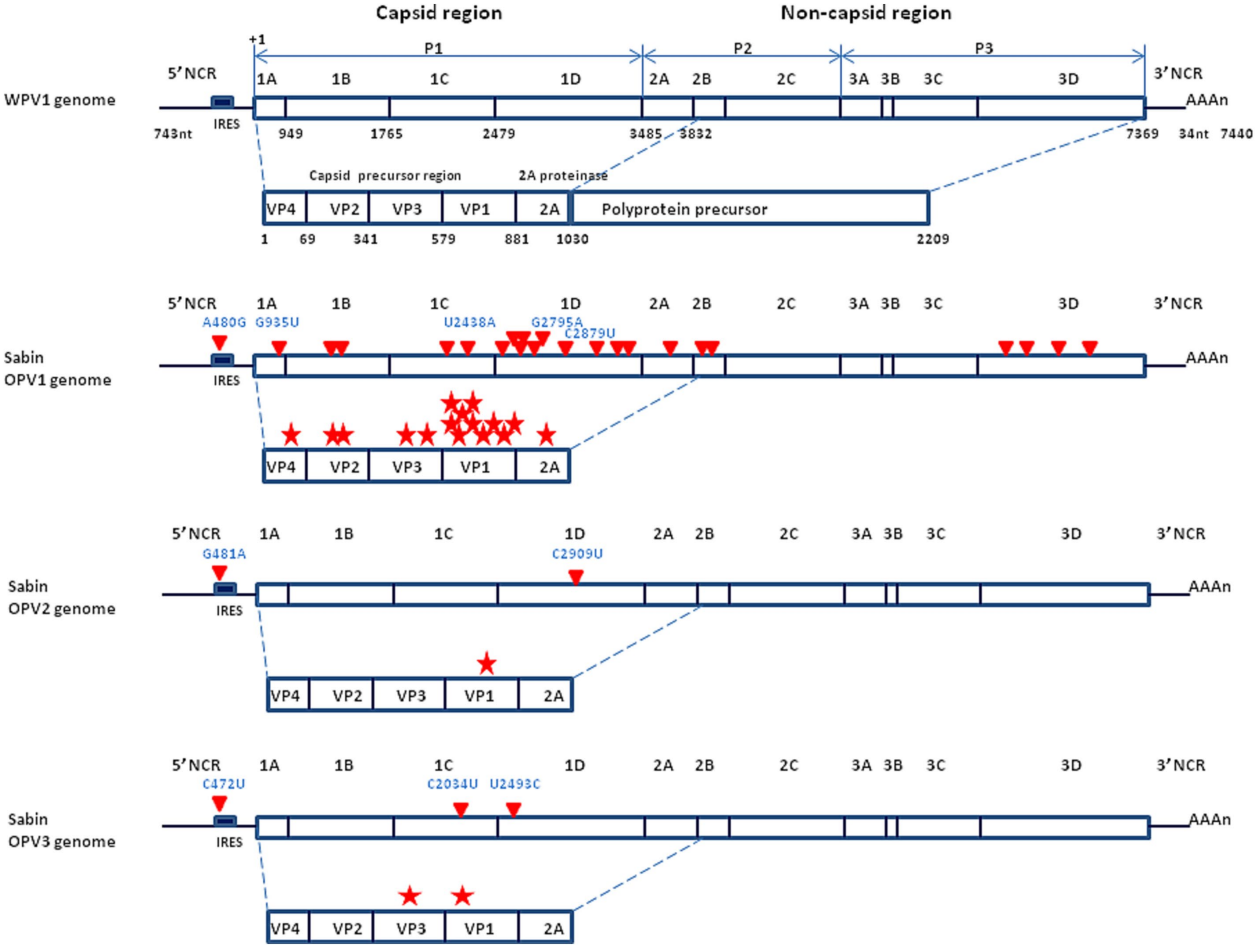
Schematic representation of the genomic organization of the wild type poliovirus (Mahoney strain) and the Sabin live attenuated poliovirus vaccine. (Upper panel) Mahoney virus. P1 is the precursor of the capsid proteins; P2 and P3 are the precurors of the non-capsid proteins. The genome segment from nucleotide 743 to 3,832 encodes a polyprotein precursor amino acids 1 to 1,030. Non-coding region = NCR (also designed UTR for untranslated region); nuclotide = nt. (Middle panel) Sabin OVP1 strain. The complete nucleotide sequence of the attenuated Sabin OPV1 compared with the WPV Mahoney strain (32), identified 57 base substitutions to be scattered all over the genome. Of these, 21 were missense mutations (red triangle) resulting in amino acid changes in a number of viral proteins, especially in the NH2-terminal half of the VP1 capsid protein, involved in attenuation (each mutation indicated by a red star). The location of the principal nucleotide substitutions leading to the attenuated phenotype is indicated in blue (A480G, G935U, U2438A, G2795A, and C2879U for the Sabin OPV1). (Lower panel) Location of the principal nucleotide substitutions leading to the attenuated phenotype of Sabin OPV2 and OPV3 (the other substitutions are not shown).

When the molecular characterization of genomes became available, it provided evidence that the WPV1, WPV2 and WPV3 and the three attenuated strains Sabin OPV1, OPV2 and OPV3, were genetically very close (17, 18, 34, 36). For example, the Sabin OVP3 (P3/Leon/12a1b) differs in nucleotide sequence from the WPV3 (P3/Leon/37) by just 10 point mutations, two of which (C472U in the non-coding region and C2034U in the structural protein VP3), confer the attenuated phenotype (37). Thus, the genetic determinants leading to poliovirus virulence are simply based on the 5′ non-coding region. A point mutation at this level is capable of changing a paralytogenic wild type poliovirus strain to a virus with attenuated phenotype. The precise mechanisms by which the capsid mutations contribute to the attenuated phenotype remain to be further documented although it can be hypothesized they are associated with changes in affinity for the CD155 receptor or effects on the stability of the capsid. The major determinant of the OPVs neuro-attenuation maps to a single point mutation located within the 5′-end internal ribosome entry site (IRES) at nucleotide 480, 481 or 472 in the case of OPV1, OPV2, and OPV3, respectively (30) (Figure 3).

The IRES mediates translation through its interaction with host cell RNA-binding proteins such as poly(C)-binding protein 1 (PCB1), PCB2, polypyrimidine tract-binding protein (PTB), the eukaryotic initiation factor 2 (eIF2) and eIF4G. The study of the interaction of the IRES of WPV3 and OVP3 with the PTB and a neural cell-specific homologue, nPTB, indicated that both PTB and nPTB proteins bind to a site directly adjacent to the attenuating mutation C472U in the 5′-end non-coding region, and that binding at this site was less efficient on the OPV3 IRES than on the WPV3 IRES, leading to a translation deficit for the OPV3 IRES (31).

## 5 When the vaccine becomes a source of pathogens

There are frequent genotypic reversions of attenuated vaccine strains that can cause poliomyelitis (these revertants are referred to as cVDPV for “circulating vaccine-derived poliovirus”). In countries that practice mass OPV vaccination, the majority of infections arise either in adults who had not been vaccinated and had come into contact with infants excreting the live attenuated vaccine, or in vaccine recipients themselves, with the most frequent circulation of OPV2 revertants (named cVDPV2) (38–41). OPV2 was estimated to cause up to 40% of all vaccine-associated paralytic poliomyelitis (VAPP) cases and 90% of all cVDPV cases (42). However, other OPVs can revert. For example, 10 paralysis cases due to cVDPV1 were reported in Madagascar in 2015.

In order to respond to the increasing number of cases of cVDPV2, OPV2 cessation was implemented globally between April and May 2016, through a synchronized switch by 155 countries from trivalent OPVs (tOPVs) to bivalent OPVs (bOPVs). According to WHO guidelines on OPV2 cessation, countries which had switched from tOPVs to bOPVs, should introduce a routine immunization schedule with an inactivated form of OPV2 (iOPV2) in order to keep the immunity against WPV2 high. Global surveillance data on OPV2 and cVDPV2 from 495,035 children with acute flaccid paralysis (AFP) in 118 countries and in 8,528 sewage samples collected between January 2013 and July 2018 in four countries with a high risk of transmission, indicated that the prevalence of OPV2 in stool samples declined from 3.9% at the time of OPV2 withdrawal to 0.2% 2 months after withdrawal (43). By testing environmental samples and stool specimens from healthy children, it was shown that all isolated viruses were related to OPVs while no WPVs were found (44). The majority of isolates belonged to OPV3. The last detection of OPV2 occurred in July 2016, 3 months after its withdrawal.

**Figure 4:**
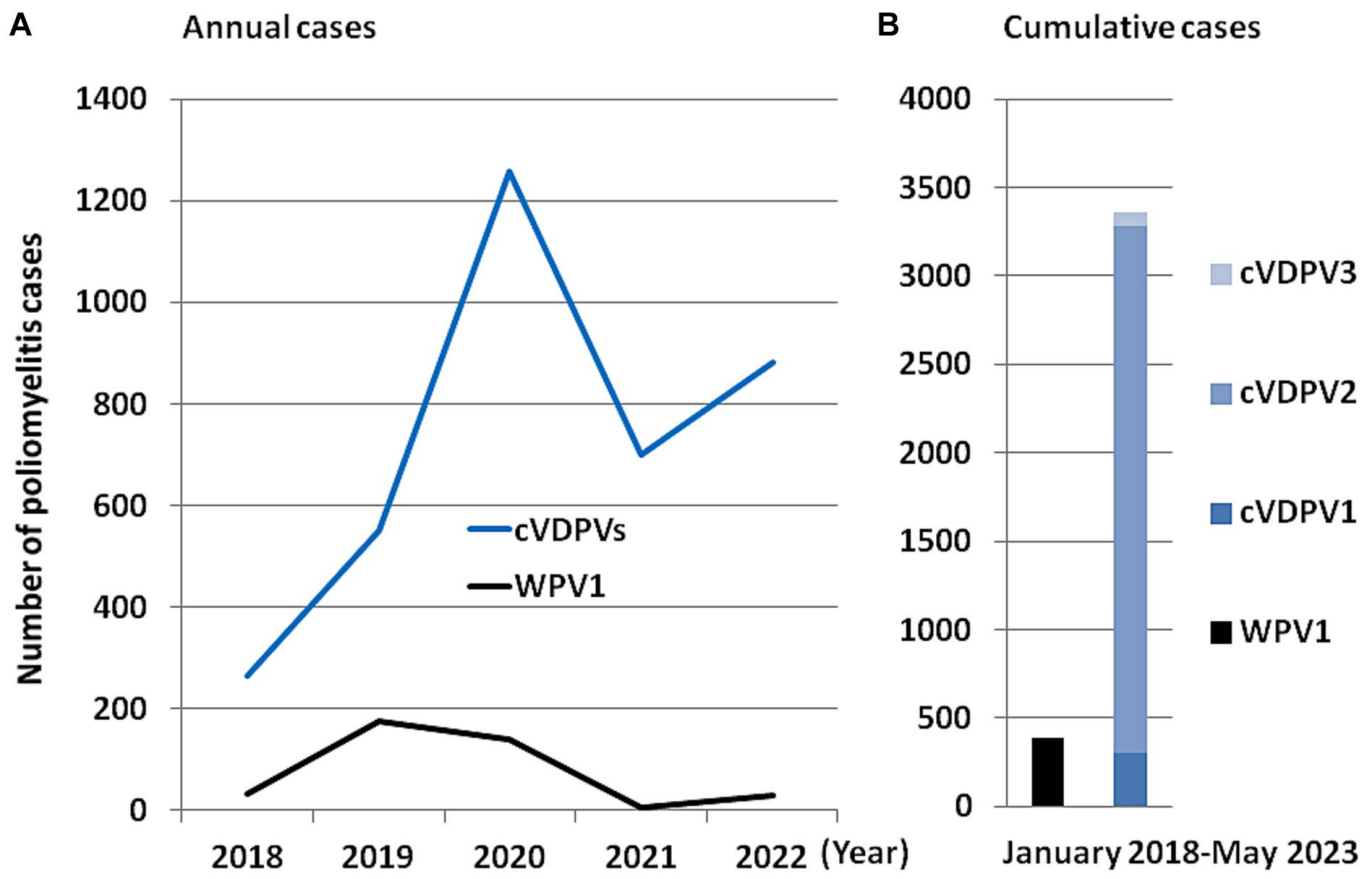
Schematic representation of the number of polio cases by comparing the cases linked to wild poliovirus and to the vaccine based on the live attenuated virus. **(A)** Number of annual cases of polio in the world that have been linked to the wild virus (currently only WPV1) and to the different cVDPVs viruses. **(B)** Number of cumulative cases of polio in the world linked to the wild virus (currently only WPV1) and to the different cVDPVs (cVDPV1, cVDPV2 and cVDPV3) viruses between January 2018 and May 2023. The values used to make this figure are those available via the ECDC (https://gis.ecdc.europa.eu/portal/apps/dashboards; accessed on June 1, 2023).

Although the objective of eradicating poliomyelitis has never been so close (polio remained endemic in only six countries in 2003; only four countries—India, Pakistan, Afghanistan and Nigeria in 2006; and three countries in 2016) and is frequently described as a success story for global health, the endgame has not been achieved and is still going on now (45, 46). In 2019, only 175 cases of poliomyelitis were reported, compared with 715 cases in 2000, and 350,000 cases in more than 125 countries in 1988 according to WHO (47, 48). According to the “Global Circulating Vaccine-derived Poliovirus (cVDPV) record” (4 June, 2019) published by the WHO, between 2015 and 2019, 50 paralysis cases due to cVDPV1 (including 10 cases in Madagascar in 2015 and 26 cases in Papua New Guinea in 2018) and 196 paralysis cases dues to cVDPV2 (including 42 cases in the Democratic Republic of Congo in 2017/2018 and 34 cases in Nigeria in 2018) and seven cases with cVDPV3, have been reported (49). In 2021, only two cases of WPV1 were recorded worldwide, one in Afghanistan and one in Pakinstan. In the weekly GPEI report issued on 19 April 2023, three WPV1 environmental samples were reported in Afghanistan, three cVDPV2 environmental samples were reported (one in Algeria and two in Burundi, respectively), and three cVDP1 human cases were reported in Mozambique (50). Notably, in 2022 paralytic cases of WPV1 outside an endemic area of WPV1 transmission (e.g., Pakistan and Afghanistan), were reported in Malawi and Mozambique (51). According to the ECDC polio dashboard sourced from the GPEI (52), in the four-month period from 1 January 2023 to 25 April 2023, the international medical authorities recorded only one poliovirus case due to WPV (one case of WPV1 in Pakistan), for 61 poliovirus cases due to the administration of OPVs, including 24 cases of cVDPV1 (12 cases in the Democratic Republic of the Congo, nine cases in Madagascar, three cases in Mozambique), 37 cases of cVDPV2 (19 in the Democratic Republic of the Congo, five in the Central Africa Republic, five in Chad, three in Indonesia; two in Benin; one in Nigeria; one in Somalia; and one in Israel). As shown in Figure 4, between 1 January 2018 and May 2023, 390 polio cases associated with WPV1 were reported worldwide while over the same period 3,442 polio cases were linked to the administration of live attenuated OPVs.

Although polio is associated with the oral-fecal route and is endemic in a few poor countries, a paper was published in September 2022 written by a former news editor of Science, Roberts, entitled “*Polio is back in rich countries, but it poses a far bigger threat to developing world*” (53). This focus on the polio risk in rich countries was related to unlikely cVDPV2 outbreaks in the United States (New York state), the United Kingdom (London) and Israel (Jerusalem), three countries which use IPV-using countries. It is unclear where and when the emergence occurred (54). One epidemiological investigation suggested that a child from Afghanistan or Pakistan who had received an OPVs at the end of 2021, had traveled to the United Kingdom while the child was still shedding the virus in stools (53). The child’ virus spread in an undervaccinated Jewish community in London (UK) and likely reverted with a few genetic changes compared to OPV2. This cVDPV2 was found to evolve slowly and was found in wastewater collected for SARS-CoV-2 surveillance. The virus went on to spread from this community to Jewish communities in Jerusalem (Israel) where it was detected in sewage during routine detection, and New York City (USA). In New York, the emerging cVDPV2 paralyzed one young man and the genetic characterization of the isolate showed the presence of 10 mutations in the critical VP1 region of the virus. There is a concern with this virus since 40% of children under the age of two lacked anti-polio immunity.

## 6 Reversion of OPV strains leading to cases of poliomyelitis

Although fully justified and effective in curbing a poliomyelitis pandemic in the 1950s at the time of pioneer Sabin’s works, this old vaccinological method of virus attenuation presents the disadvantage of allowing an RNA virus to replicate in humans, where it can mutate and certain mutations of which can contribute to a return to a virulent virus phenotype. Evidence that attenuating mutations can revert or be suppressed in virulent isolates found in vaccine-associated cases of poliomyelitis, has long been reported (55). Extended replication and the mutation of the OPV can lead to cVDPVs being defined as 1% divergence from the parent strain for OPV1 and OPV3 and 0.6% divergence from the parent strain for OPV2 (56). Such viruses can also be shed from healthy recipients (35). Although impossible to ignore, these disturbing results did not undermine the determination of infectiologists to continue mass vaccination, insofar as the benefit/risk ratio at the level of the general population was favorable to the continuation of vaccination with OPVs. Ninety per cent of revertants isolated belonged to the OPV2 and OPV3 types. One important site which is critical for attenuation lies in the 5′ non-coding region of the genome of each of the three OPV strains, at nucleotide 480 in OPV1, 481 in OPV2 and 472 in OPV3, respectively. The search for the revertant genome in 14 isolates from VAPP strains and in 16 strains isolated from healthy vaccinees highlighted reversion in all 14 isolated from VAPP strains (either cVDPV2 or cVDPV3), while reversion was found in 3 strains (cVDPV1) isolated from healthy vaccinees (57). Another study reported that for the type 2 cVDPV revertants, the amino acid residue at position 143 of VP1 was found to be a valine, a threonine, an asparagine or a serine in the revertants, while it was an isoleucine in OPV2. Such revertants at positions 143 of VP1 are associated with VAPP. For the type 3 cVDPV, a serine at position 91 of VP3 in the revertants in place of a phenyl alanine in OPV3 is frequently associated with another mutation at amino acid residue 54 in VP1 (58). A study of different complete genome sequences of cVDPV1 (also named iVDPVs when isolated from immmunodeficient patients) isolated during the chronic infection of an immunodeficiency patient from Taiwan over a period of 30 months starting in 2001, showed that divergence of separate lineages began at the start of the infection and continued for at least 18 months (59). The different lineages which emerged harbored 2.43% to 3.53% variation in VP1 compared to the parental strain, and showed deletions and nucleotide substitutions in 5′-end NCR and recombination across and within lineages, suggesting that the cVDPV1 had established separate sites of replication within the gastrointestinal tract but the detection of recombinants indicated that the tissue compartmentalization was incomplete and that viruses from different lineages can co-infect the same cell and recombine. Similarly, intratypic recombination between co-evolving lineages of cVDPV2 has been reported (60). Before the decision in 2016 to “switch” from tOPVs to bOPVs, circulation of cVDPV2 was documented in Myanmar and Nigeria in 2015, and in Guinea in 2015 and 2016, while cVDPV1 outbreaks were reported in the Lao People’s Democratic Republic, Madagascar, and Ukraine, and there were no ongoing outbreaks of cVDPV3 (45).

## 7 Mechanisms of reversion and recombination in RNA viruses

Like other RNA viruses, polioviruses are evolving according to the quasi-species (mixtures of closely related variant genomes termed mutant spectra) model characterized by continuous genetic variation as a result of a high rate of base misincorporation by the viral RNA-dependent RNA polymerase (61, 62). Although different models could be hypothesized, the common model of quasi-species evolution considers that mutations are not preexisting but instead are acquired after infection. However, depending on the viral concentration of the infectious source, according to Domingo and Perales (62) “*this viral population can include a reservoir not only of genotypic but also of phenotypic variants, conferring upon the population some adaptive pluripotency*.” RNA virus mutation rates are strongly influenced by host-encoded factors, and an extensive mutational bias may be introduced by the host Apolipoproteins B mRNA editing enzyme, catalytic polypeptide-like (APOBEC), a group of RNA cytidine deaminases which targets a dinucleotide (the edited base and the −1 base; e.g., APOBEC-1 the expression of which was found in intestinal cells, deaminated cytidine in the context of 5′-AC-3′ while most other APOBEC are regarded for their ability to target dsDNA rather than RNA) and/or the adenosine deaminase acting on RNA (ADAR; a dsRNA-dependent adenosine deaminase capable of converting adenosine to inosine) which triggers an A to G base substitution (63–67). However, to our knowledge, this mechanism supporting rapid evolution of RNA viruses has never been investigated for PVs. The misincorporation of nucleotides may be non viable, neutral or have a drastic effect on the phenotype of the virus in terms of replication or pathogenicity. Under positive selective pressure from the host, spontaneously generated mutations can be selected, leading to the emergence of variant viruses able to escape the host’s defense mechanisms (68).

Recombination can mediate adaptability and virulence (69, 70). Two mechanisms can lead to chimeric genomes: “breakage and joint” (in which the genetic sequence of one parental genome is cleaved by nuclease and re-ligated with a sequence originating from a different parental genome) or “copy choice” (in which the nascent RNA strand switches template strand during genome replication). Evidence that template switching takes place predominantly during negative strand synthesis (RNA synthesis required) leading to the emergence of chimeric poliovirus suggests that chimeric poliovirus are produced as the result of the copy choice mechanism (71–74). The copy choice mechanism predicts that the frequency of recombination events should parallel the intracellular concentration of the viral RNAs (74). Among the different types of recombinations (either “homologous,” which occurs at homologous nucleotide sequences or “aberrant homologous” with imprecise crossing over leading to deletions, duplication or insertion; or nonhomologous), homologous recombination is effective in regions of high (>80%) homology (73). However, crossovers leading to insertion or deletion have also been documented (75, 76). Deletion may sometimes generate defective interfering viral particles.

## 8 Loss of OPV attenuation via base misincorporation or recombination?

**Figure 5:**
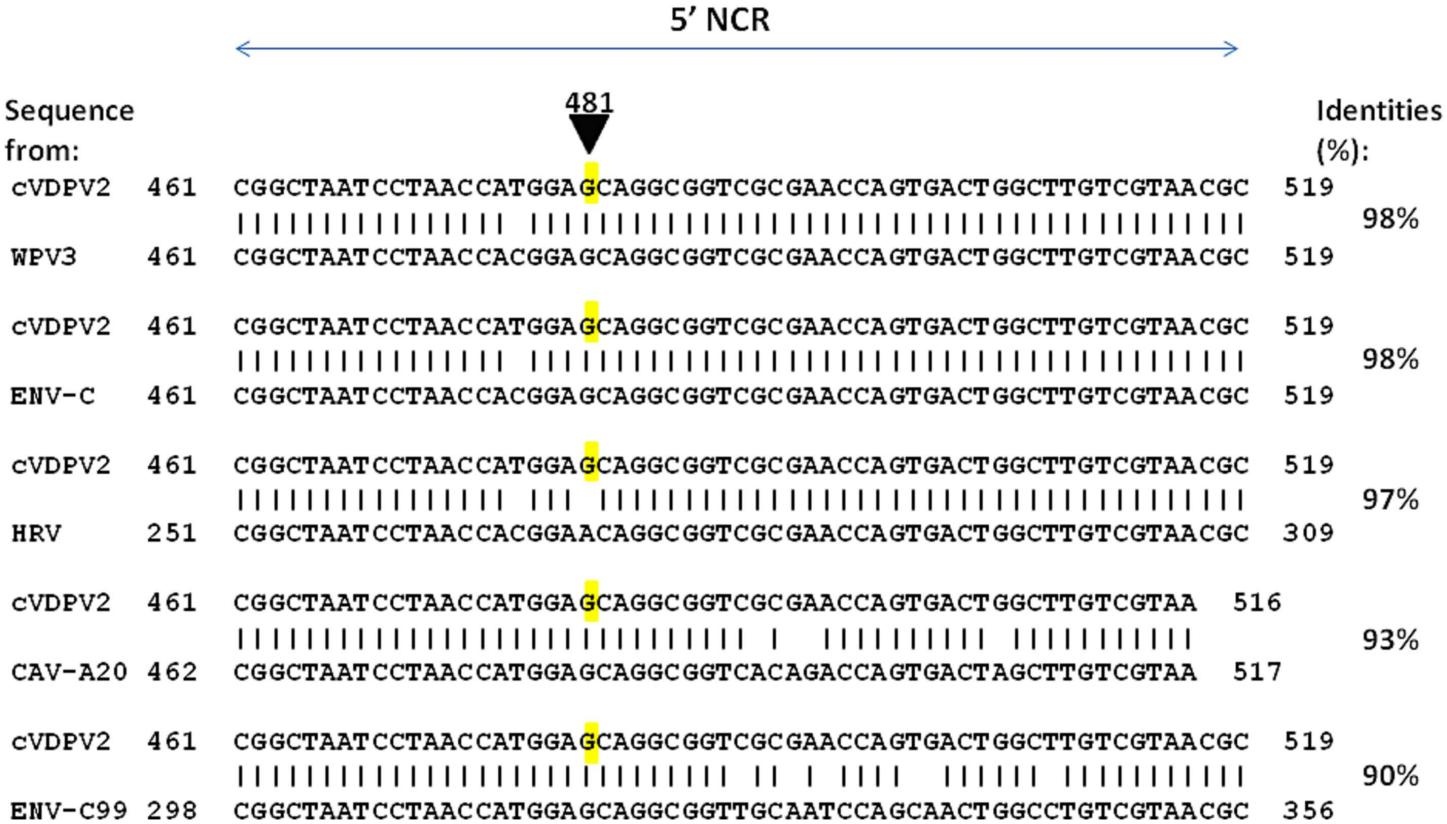
Representative examples of enterovirus sequences showing high percent identity with the nucleotide sequence 461–519 of cVDPV2 revertant. The nucleotide sequence surrounding the position 481 from an OPV2 which has loss OPV2 attenuation (cVDPV2) was blasted against the complete Base NR database to search for possible identity with other enteroviruses. Several sequences from enteroviruses contain a G within a sequence of high similarity with cVDPV2, were identified including WPV3 (WPV3 ISR_1976-12b; GenBank accession OP137305), ENV-C (Enterovirus C strain Polozj-3; GenBank accession MZ546188), CAV-A20 (Coxsackievirus A20 strain IH Pool 35; GenBank accession AF465514), ENV-C99 (Enterovirus C99 strain MAD-3185-2011; GenBank accession OK570208). Notably, a sequence from rhinovirus, HRV (Antwerp rhinovirus 98/99 isolate 99028352; GenBank accession DQ316308), shows 97% identity with cVDPV2 but contains an A481 as for the OPV2 strain.

The base misincorporation rate of polioviruses during their random genetic drift process was estimated to be in the range of 10^−5^ to 10^−4^ (28, 77) and about 10^−2^ substitutions per site per year (78). The capsid region appears to be less genetically stable than the region encoding non-structural proteins (79). Next to base misincorporation another likely important mechanism for the generation of polioviruses diversity is recombination, which occurs during the replication of the virus (80). It has long been considered that recombination may not be essential to the phenotypic reversion of OPVs, first because the main determinants of the attenuation of all three OPVs strains map to 5′-NCR and capsid region sites and most of the observed recombination sites map to the non-capsid region and second because recombination with viruses from the enterovirus-C species (ENV-C) normally occurs during the circulation of WPVs (81). Indeed, one elegant publication suggests that in several lineages, substitution leading to a loss of OPV attenuation could be obtained via recombination rather than via mutation (82). To further explore this possibility, we conducted a similar search using BLAST against the NR database at the NCBI website (unpublished data). We focused on the sequence surrounding position 481 from an OPV2 strain that exhibited OPV2 attenuation. The purpose was to identify potential similarities with other enteroviruses. As shown in Figure 5, several sequences from enteroviruses contain a G within a sequence of high similarity with cVDPV2, suggesting recombination between OPV2 and WPV3 or other enteroviruses (Enterovirus type C, Coxsackievirus A20) could have been involved in the reversion of OPV2 to a pathogenic phenotype by a mechanism in which the RNA polymerase involved in RNA synthesis from one RNA template molecule pauses within the region of intermolecular base-pairing (intermolecular duplexes) and jumps to a homologous site in a second RNA molecule.

This simple observation suggests that recombination events causing loss of OPV attenuation may be more frequent during phenotypic reversion processes than previously estimated. These events may accelerate the evolution of polioviruses by swapping entire genetic units of different strains of polioviruses or even of poliovirus strains and other enteroviruses with sequences closely related to polioviruses.

## 9 Intratypic and intertypic recombinant polioviruses

It has been estimated that 10% to 20% of the viral genomes undergo recombination in a single growth cycle (28). Recombination was demonstrated as soon as noncovariant markers were used to label the parental strains with the characterization of chimeric genomes between two WPV1 (83). Moreover, crossovers during intertypic poliovirus recombinations are non-randomly distributed (84). Some isolates may be recombinants in which the capsid proteins derive from one serotype, while the backbone genome belongs to another serotype. Evidence of serial recombination was found among the cVDPVs such as isolates from Hispaniola (four different recombinants detected) (85) or Egypt (22 different non-capsid region recombinants and two different 5′-end NCR recombinants were detected) (86).

Recombination among WPVs has been well documented (73, 87–89). The analysis of RNA recombinants showed that intertypic RNA recombinations are not site-specific and do not require extensive similarity between the recombining parents at the crossover site with a probable copy choice mechanism for RNA recombination, in which the viral RNA polymerase switches templates during negative strand synthesis (73, 90). The intertypic recombinants are usually found about 10 days after vaccination, essentially in vaccinees excreting OPV3. The WPV3F isolate from Finland showed high homologies with the other WPV3 only in the P1 region, while it turned to be genetically closer to WPV2 in other portions of its genome indicating intertypic recombination (91). It has been estimated that the intratypic recombination frequency for the entire genome is approximately 15% (92). Recombination between intertypic WPV strains, which have 85% nucleotide identity, has been considered to occur at a 100-fold-lower frequency than intratypic recombination between completely homologous parental serotypes (73).

Similarly to what is observed between WPVs in terms of recombination, such a viral dynamic is obviously also observed with OPVs. Recombination events between polioviruses were evidenced by isolating viruses showing intertypic chimeric non-capsid sequences from children exposed to the trivalent oral poliovirus vaccine (tOPV) (93, 94). OPV strains rarely spread beyond the close contacts of vaccine recipients (95). Recombinant virus with their 5′-end inherited from OPV3 and their 3′-end inherited from OPV2 have been isolated from healthy vaccinees. In some vaccinees, the OPV3/OPV2 recombinant viruses can further recombine and the insertion of sequences from OPV3 or OPV1 at the 3′-end of their genome can occur (58). Between August 2011 and February 2012, an outbreak of AFP was caused by cVDPV2, the genome of which consisted of a mutant (mut) of OPV2 (0.7% to 1.2% difference in VP1 compared with OPV2), a reversion in A481 in the 5′-end of the genome, and three crossovers leading to the insertion of OPV3 sequences into the cVDPV2 backbone (96). Yan et al. (96) reached the conclusion that due to the risk of cVDPV2, the OPV2 should be removed from the trivalent OPVs formulation. Although Sabin OPVs is considered the most effective and safest polio vaccine currently used, it can be responsible for polio cases (97–99). It has been estimated that 0.84 cases of poliomyelitis per million recipients can develop subsequently to vaccination, mainly with OPV2 and OPV3 (37, 57, 97, 100).

## 10 Enteroviruses other than polioviruses can cause a poliomyelitis-like disease

The viruses which cause poliomyelitis leading to paralysis belong to the genus Enterovirus, family Picornaviridae, order Picornavirales, Class Pisoniviricetes, phylum Pisuviricota, kingdom Orthornavirae, realm Riboviria. The Picornaviridae family contains 147 species. Currently the genus Enterovirus contains 12 enterovirus (ENVs) species (enteroviruses, A-L according to the ICTV Virus Taxonomy; https://ictv.global/report/chapter/picornaviridae/picornaviridae/enterovirus accessed on July 8, 2023). The three serotypes of human-specific PVs belong to the enterovirus C species. The family of Picornaviridae also contains serotypes of coxsackie A viruses (CAVs) and coxsackie B viruses (CBVs), serotypes of echoviruses (EVs), and three serotypes of rhinoviruses (A, B, C), reflecting a wide range of evolutionary divergence (101, 102). Enteroviruses are widespread in the world and some are at the origin of a number of infection in humans (103). It has been recognized that paralytic syndromes which closely resemble poliomyelitis may also develop in association with non-polio enterovirus infections such as coxsackievirus A7 (CAV-A7) and CAV-A9 (104, 105), Echovirus 11 (EV-11) (106), human enterovirus (ENV) 70 and EV-71 (107, 108) and ENV-D68 (109). Between 1969 and 1973, outbreaks of ENV-71 occurred in California (108). Large epidemics of paralysis due to ENV-71, occurred in Bulgaria in 1975 (more than 700 cases, 149 patients developed paralysis and 44 others died) and Hungary in 1978 (110). Between 1976 and 1979 in the US over 50% of all reported cases of paralytic diseases were due to non-polio enterovirus infections (111). The EV-71 is endemic in the Asian-Pacific region and recognized as a frequent cause of epidemics of hand-foot-and mouth disease (HFMD) associated with severe neurological complications and high death rates among children <5 years of age (112–115). During the Indian ENV 70 acute hemorrhagic conjunctivitis epidemic in 1981, patients were found to have neurological complications (polio-like syndrome pre-paralytic, paralytic, and post-paralytic stage of slow recovery) after a variable latent period (107, 116). Notably, cases of acute flaccid paralysis were reported in several independent epidemiological clusters of children during an outbreak of ENV-D68 in the United States in 2014 (109). These examples illustrate the fact that several enterovirus, chiefly ENV-D68 and ENV-A71, have emerged as a cause of severe respiratory disease and AFP, due to recent genetic virus evolution (117, 118).

The rate of asymptomatic carriage of enteroviruses in humans is known to be considerable. For instance, in a study conducted in the Netherlands in the general population including children and adults, enteroviruses were detected by real-time PCR in stools from 33 (3.0%) of 1,100 asymptomatic people [compared to in 42 (3.1%) of 1,340 patients with symptoms of gastroenteritis, which was not significantly different] (119). Also, in a study conducted in Nigeria among children younger than 10 years of age, enteroviruses were isolated in culture from stools collected from 42 (6%) of 756 asymptomatic children [compared to in 96 (10%) of 966 children] (120). Moreover, long-term asymptomatic enterovirus carriage has been reported among immunocompromized individuals (121). These viruses can potentially be a source of genetic material for recombination with polioviruses.

## 11 Recombinant between polioviruses and other enteroviruses

Although most vaccine-derived recombinants were produced by genetic exchange between the three vaccine strains (94), some recombinants arose from exchange with WPV and other enteroviruses (100). Some genome regions of polioviruses may be interchangeable with those from different enterovirus species, as demonstrated by the *in vitro* production of viable interspecies recombinants (122–126). It is quite obvious that for recombination to occur between polioviruses and other enteroviruses, the first requirement is that the two viruses infect the same cell. Moreover, the frequency of recombination is likely to be a function of the total number of mixed infections, which follows from the combined carriage rates of polioviruses and species of ENV-C. The host range of poliovirus is determined by the CD155, a transmembrane glycoprotein composed of three extracellular immunoglobulin-like domains (a membrane-distal V-type domain that binds poliovirus followed by two C2-type domains), the physiological function of which is to serve as an adhesion molecule acting on cell proliferation and migration, and a recognition molecule for natural killer (NK) cells (127, 128). Most of the immunological effects of CD155 are mediated by its interaction with the DNAX accessory molecule-1 (DNAM-1) (also called CD226) or CD96 on the surface of leukocytes. The CD155 poliovirus receptor is expressed in a large number of tissues including tissues from the gastrointestinal tract (it has high expression in the stomach, colon and rectum and intermediate expression in the small intestine and duodenum) as well as the CNS (it has high expression in the cerebral cortex, intermediate expression in the cerebellum) (129). It is expressed at the surface of intestinal epithelial cells and on M cells as well as neurons. The receptor used by coxsackieviruses is the coxsackievirus and adenovirus receptor (CXADR also named CAR) which also belongs to the immunoglobulin superfamily of cell adhesion molecules and which is expressed in most tissues (130). Enterovirus B infection, including coxsackie B viruses, also requires the human neonatal FC receptor as the uncoating receptor, whose expression may be more restrictive than that of CAR (131). Several molecule can stimulate enterovirus 71 attachment to target cells, including scavenger receptor B2 (SCARB2), P-selectin glycoprotein ligand-1 (PSGL-1), sialylated glycan, heparan sulfate and annexin II (Anx2). SCARB2 plays critical roles in attachment, viral entry and uncoating, and it can facilitate efficient EV71 infection (132). In contrast to CD155 and CXADR, SCARB2 lacks an immunoglobulin-like fold (133). SCARB2 is expressed in a variety of tissues including the intestinal epithelium and neurons (134). Receptors for different enteroviruses are often co-expressed on the same cells, facilitating the recombination.

**Figure 6:**
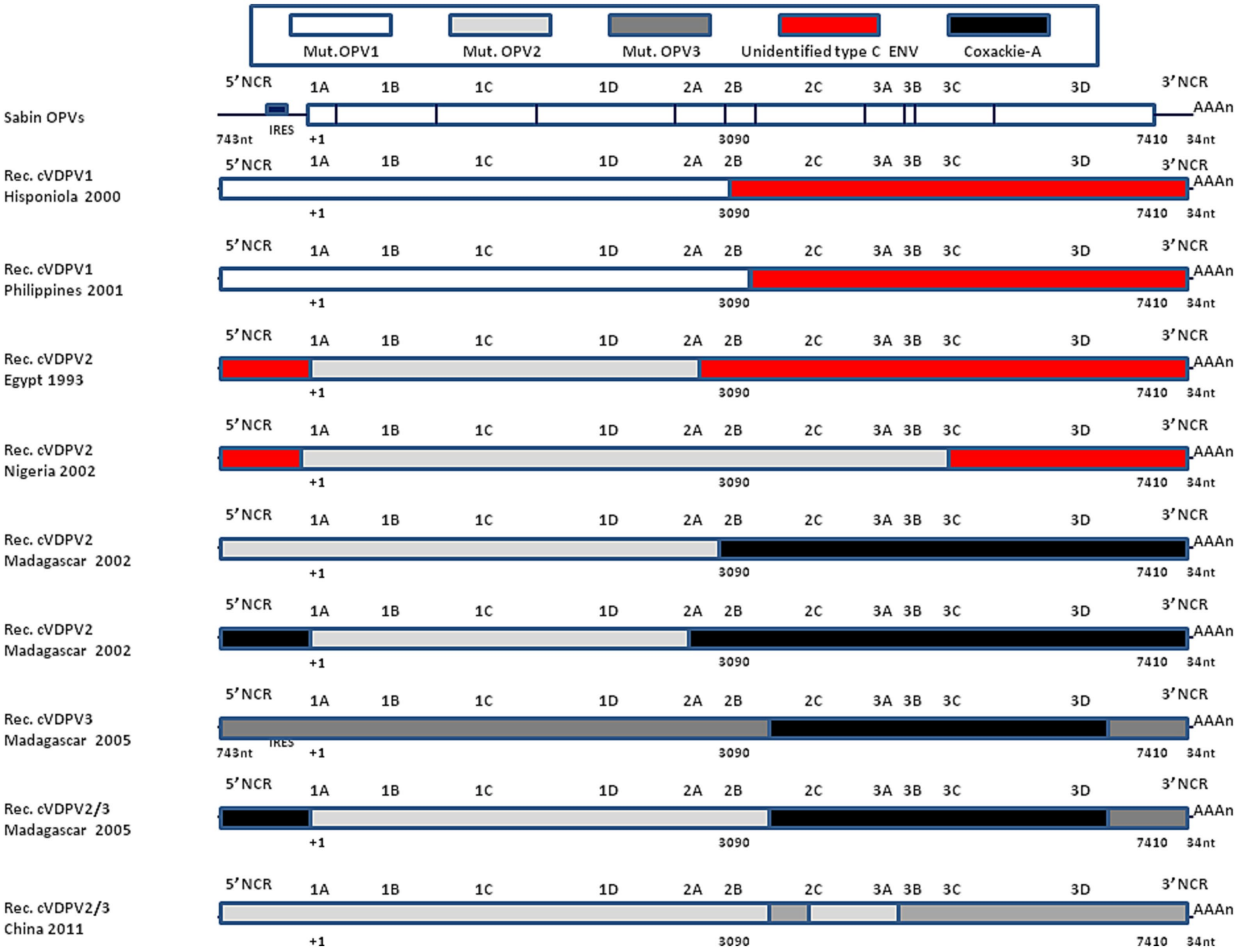
Recombinant cVDPVs. Schematic representation of the genomic organization of some cVDPVs that have been isolated during different outbreaks between 1982 and 2005 in various geographic areas (Hisponiola, the Philippines, Egypt, Nigeria, Madagascar, China). The outbreaks corresponded to the emergence of mutated OPV1, OPV2, or OPV3, which had recombined with either human enteroviruses (ENV-C) including Coxackie A (CV-A11, CV-A13, or CV-A17). Data are adapted, modified and/or summarized from references (84, 85, 96, 136, 139). For example, one of the cVDPVs isolated in Madagascar in 2005 was a mosaic genome composed by a mutated OPV2 with a recombinant 5′-end derived from Coxsackie A-13, a sequence encoding non structural proteins derived from Coxsackie A-17 and a 3′-end from OPV3. More recently, a strain was isolated in London, UK, that showed a recombinant genetic structure between cVDPV2 (with reversion of the two main OPV2 attenuation mutations) and an unidentified enterovirus C species with a crossover point at nucleotide 5,139 (142), not shown.

Johnson et al. (122) reported that a virus designated PCV1 was a recombinant with a coxsackievirus 5′-end NCR sequence with a 4-base deletion that modified its temperature sensitivity phenotype. Similarly, the insertion of a 405-nucleotides fragment from the 5′-end NCR of the coxsackie CBV3 lead to an infectious recombinant virus with modified temperature sensitivity phenotype (125). Replacing the 3′-end NCR of WPV3 by CBV4 lead to replicating recombinant virus (124). Replacing the 2A-encoding sequence of WPV by coxsackie CBV4 or rhinovirus HRV2 also yielded viable virus in transfected cells (123), while replacing the 2B of coxsackie CBV3 by the 2B from WPV gave a recombinant virus which failed to replicate although another chimeric genome that expressed a hybrid 2B protein consisting of the amino-terminal one-third of WPV and the remainder of CBV3 yielded viable viruses (126). Bessaud et al. (135) constructed 29 genomes containing the OPV2 capsid-encoded sequence and other sequences from OPV2 or from non-poliovirus type C enteroviruses. Most genomes were functional, being able to replicate *in vitro* but differed in their plaque size and temperature sensitivity. The observation that an *in vitro* chimeric genome between WPVs or OPVs and other enteroviruses yield viable recombinant viruses is corroborated by the dynamic of WPV and OPV recombination *in vivo* (136). This evolution dynamic can be even faster in immunodeficient individuals (137, 138). OPVs replicate in the gastrointestinal tract mimicking natural WPV infection. During the intestinal phase of poliovirus replication, it is common for the same enterocyte to be subjected to a simultaneous infection by another enterovirus (e.g., coxsackieviruses which share homologies with polioviruses), which can lead to genomic recombination resulting in the production of chimeric viruses (73). The genomic analysis of polioviruses that circulated between 1988 and 1993 in Egypt causing 30 cases of poliomyelitis, indicated that such viruses were cVDPV2 and the complete genomic sequences of an early (1988) and a late (1993) cVDPV isolate revealed that their 5′-end NCR and 3′-end NCR sequences were derived from other species C enteroviruses (85). In 2001, cVDPV1 were isolated from three AFP patients and one contact case in the Philippines. The complete genomic sequencing of these four cVDPV isolates revealed that the capsid region was derived from the OPV1 strain (which most likely originated from administration of a dose of OPVs in 1998/1999) but that most of the non-capsid region was derived from a crossover at nucleotide position 3,949 with an unidentified enterovirus unrelated to the oral poliovirus vaccine (OPV) strains (139). The analysis of a large poliomyelitis outbreak in Nigeria between 2005 and 2011 with an estimated to 700,000 cases was due to a cVDPV2. A dominant cVDPV2 lineage expanded rapidly in early 2009, fell sharply after two vaccination campaigns with tOPVs in mid-2009, and gradually expanded again through 2011. The two major determinants of the attenuation of the OPV2 vaccine strain (A481 in the 5′-end NCR and VP1-Ile143) had been replaced in all cVDPV2 isolates, and most A481 replacements occurred by recombination with other non-polio enteroviruses (140). More recently, a cVDPV2 (the two key attenuating mutations A481G in the 5′-end NCR and Ile143Thr in VP1 had reverted) was isolated from a patients with AFP admitted to hospital in Spain from Senegal. In depth analysis of the genome revealed a chimeric structure between cVDPV2 and an unidentified non-polio-enterovirus type C strain with a crossover in the protease 2A region. This isolate corresponded to a strain that circulated in 2020–2021 in Senegal (141). In 2022, a cVDPV2 was isolated in London sewage (142). Figure 6 illustrates the genomic organization of some cVDPVs that have been isolated during different outbreaks in different countries.

## 12 The WHO emergency use listing strategy

Upon the initiative of the WHO, the United Nations International Children’s Emergency Fund (UNICEF, currently renamed “United Nations Children’s Fund”) and Bill & Melinda Gates Foundation (BMGF), a major endeavour was launched to develop a more genetically stable version (genetically modified) of the monovalent Sabin oral polio vaccine type 2 (mOPV2) in order to stop outbreaks of WPV2 more sustainably. The first-in-human clinical trial with the “novel” OPV2 (nOPV2) was conducted in 2017 in Belgium (143). The nOPV-2, derived from a modified OPV2 that includes five modifications in the genome including change in the 5′-NCR, the capsid coding region (P1) the non-structural protein 2C and the polymerase 3D^pol^, was found to be safe (severe adverse events were infrequent and considered to be causally associated with vaccination and similar to mOPV2 historical control), lower shedding than mOPV2, well tolerated and immunogenic in children and infants (144–146). The nOPV2 is envisioned as a major player in the final stage of polio eradication. To date, about 600 million doses of the nOPV2 (supplied by BioFarma, Bandung, Indonesia) have been used across 28 countries for outbreak response (and not in routine programs), in line with the Emergency Use Listing (EUL) recommandations of the WHO.

Although the effectiveness of nOPV2 in stopping paralytic outbreaks seems to be comparable with Sabin mOPV2, with a lower risk of seeding new outbreaks compared to the Sabin mOPV2, the risk of recombination with other enteroviruses remains a source of concern. As fairly acknowledged by A. Bandyopadhyay from the BMGF in a paper from the GPEI (147): “*I must clarify that significantly higher genetic stability and lower risk of reversion to neurovirulence compared to Sabin mOPV2 does not mean nOPV2 has no risk of reversion. We’ve seen, very recently, evidence of reversion of public health significance in the Democratic Republic of the Congo (DRC), where two separate emergences of circulating* var*iant poliovirus type 2 of nOPV2 origin have been detected, likely derived from double recombination events with human species C enteroviruses. Such recombination events and reversions resulted in seven paralytic cases in the DRC and neighboring Burundi. In separate events, we also saw the detection of reverted and recombinant poliovirus variants of nOPV2 origin in two cases of paralytic polio in the Central African Republic in February 2023 and in an environmental sample collected in Uganda in February 2022. With the much wider scale of nOPV2 use in areas of very limited background intestinal immunity for type 2 poliovirus, we may pick up more of these rare reversions going forward.*” This illustrates how fragile the effort is to curb circulating cVDPVs, which continue to pose a threat to under-immunized communities and to eradication of polio worldwide. Because polioviruses are known to recombine with other human enteroviruses, and often only the capsid is maintained, a strategy of codon-deoptimization (CD) based on synonymous codon substitutions was choosen by Konopka-Anstadt et al. (144), since it was demonstrated that synonymous codon substitutions increasing the CpG and UpA dinucleotide pair in the capsid of OPVs reduce replicative fitness. The engineered vaccine (nOPV2-CD) which had modified 40% of all possible CpG sites in the capsid region also featured a modified 5′-end NCR and was shown to retain similar antigenicity with OPV2. Greater genetic stability is a key common goal in redesigning nOPVs. Changes introduced in the genome of nOPV2 backbone for stabilization served for the development of nOPV1 and nOPV3 live attenuated vaccines. The candidates were generated by replacing the capsid coding region of nOPV2 with that from Sabin 1 or 3 (148). There are plans from BMGF to launch nOPV1 and nOPV3 formulations into phase II studies in 2023 with the objective of replacing OPV1 and OPV3.

The genetic instability of poliovirus vaccine emerged as a concern even in rich countries after the recent cVDPV2 polio case in New York (53) for several reasons: the variant cVDPV2 was found in wastewater, there is insufficient routine immunization (about 40% of children under the age of two are not fully vaccinated against polio), and the nOPV2 has not yet been approved to be used in the USA (in the USA, the IPV vaccine is used to preventing paralysis but is not as good as OPVs/nOPVs at preventing outbreaks). In the USA WPVs had most likely been eliminated by the early 1970s (the USA was declared free of indigenous WPV in 1994) and the country switched from OPVs to the safe and effective IPVs. According to the Center for Disease Control Atlanta, two doses of IPVs are 90% effective against paralytic polio and three doses of IPVs are 99% to 100% effective. However, several immunologists consider that the IPVs do not induce the robust mucosal immunity required to prevent viral circulation (149, 150). Moreover, US children are currently under-vaccinated. Delaying the cVDPV2 outbreak response to wait for nOPV2 could considerably increase the expected number of cases, as hypothesized (151). There could also be major risks of removing OPV vaccination from countries that are relying on it to prevent the re-establishment of endemic transmission of live polioviruses. In addition, it has been reported that OPVs confer temporary non-specific protective immunity against other pathogens unrelated to poliovirus such as influenza virus, human immunodeficiency virus and severe acute respiratory syndrome coronavirus 2 (SARS-CoV-2) (152–155), which can be advantageous when these viruses circulate at high frequency in populations.

## 13 Interspecies circulation of polioviruses

WPVs are highly contagious viruses which are capable of virtually infecting the entire population in endemic areas with poor sanitation and hygiene, most children being infected by the fecal-oral or oral-oral route within the first year of life. The host range of polioviruses is determined by the CD155 receptor, an important cell–cell adhesion molecule involved in the transendothelial migration of leukocytes through interaction with CD226 (122, 155, 156). CD226 is found on the microfold (M) cells of Peyer’s patches in the gastro-intestinal tissues of humans, higher primates, and Old World monkeys (157). Polioviruses can infect chimpanzees and gorillas, as well as more distantly related anthropoids, such as colobus monkeys (158–162). Paralytic poliomyelitis can be experimentally induced in macaque species (rhesus, cynomolgus, and bonnet) by oral WPV1 infection (161). Monkeys have largely been used to evaluate the neurovirulence of poliovirus strains (161–163). The host range of polioviruses is considered to be restricted to humans and simians as a consequence of receptor polymorphism in mammals (160). However it has been known since the early 1960s that poliovirus can initiate replication in resistant cells as long as measures are taken to circumvent the barrier to infection. Chicks are naturally insusceptible to poliovirus infection, however, intracerebral inoculation of poliovirus RNA induces virus production (164). It has been reported that chick embryo cells or hamster embryo cells previously exposed to poliovirus type 1 and then exposed to irradiated Sendai virus as a fusion inducing agent (which induce cell fusion and multinucleated giant cells), leads to the replication of poliovirus (165). Murine cells can become susceptible to poliovirus infection after they have been transfected with a DNA construct expressing the human CD155 receptor (166, 167). Infected cells show cytopathic effects within 4 to 6 h, and release up to 10,000 newly infectious virus particles upon cell lysis.

Outbreaks of polioviruses have been reported in captive and wild non human primates in Africa. Accidental exposure to infected laboratory workers has led to poliovirus infections in chimpanzees and gorillas since the 1940s (168). In 1966, six chimpanzees at the Gombe Stream National Park in Tanzania died from a polio-like virus, and six others were paralyzed for life, shortly after a polio epidemic swept through neighboring human settlements (169). In 1982, an outbreak of WPV1 was documented in three captive black and white colobus monkeys from Kenya (158). WPV1 was isolated from the feces, spleens, kidneys, lungs and central nervous systems of affected animals. Notably, our team reported in the Lésio-Louna-Léfini Nature Reserve (Republic of Congo) the presence of a new enterovirus C, genetically related to WPV1, WPV2 and coxsackievirus (CAV-A13) in a male gorilla with clinical symptoms of facial paralysis. This case of facial paralysis led to further investigation of enteroviruses in gorilla and humans in contact with these non-human primates (NHP). Enteroviruses were detected in the feces of 29.4% of gorilla (including wild and human-habituated gorillas) and feces of 13.15% of humans (including local residents and eco-guards) (170, 171). Moreover, another study conducted by Harvala et al. (172) in Cameroon and the Democratic Republic of Congo found evidence for the circulation of genetically divergent variants of enteroviruses in apes and monkeys. They found enterovirus RNA in eight of 58 chimpanzee samples (13.8%), one of 40 bonobos samples (2.5%) and five of 40 gorillas samples (12.2%). This study also highlighted that one strain (EV-A89) found in a chimpanzee sample was also shared with the local human populations. A newly identified enterovirus EV-A119 found in NHP also circulated in humans in the same area. In addition, various enteroviruses are hosted by African NHP. Notably the ENV-C99 was found in a captive chimpanzee (in the Republic of Congo), associated with AFP (173). Coxsackievirus CAV-A13 and CAV-A24, Echovirus EV-15 and EV-29, and enterovirus ENV-B82 and EV-A119, were found in chimpanzees and gorillas in Cameroun and circulate between humans and NHP (174). Moreover, other ENVs were reported in NHP, including simian type A and type B ENV such as the EV-B112 in chimpanzees and ENV-B113 in mandrills (175). The simian ENV-B107 and ENV-90 were previously found in humans. This suggests that in regions with a tropical or subtropical climate, where there are large numbers of NHP and poor sanitation and hygiene conditions (or, worse, if NHP are consumed in local human food), there is a risk of WPV outbreak in humans from contact with infected NHP, which could be a “reservoir” of polioviruses. In addition, NHP can be infected by OPVs and become a source of reintroduction of variant polioviruses and/or recombinant (with enteroviruses) into humans (176), even if the region has been declared polio free.

## 14 Discussion: implementing a new strategy to fight against polioviruses

The Sabin OPV provides long term protection against WPVs through durable humoral immunity (56). In immunocompetent individuals, the risk of vaccine-associated paralytic poliomyelitis (VAPP) is very low. The cellular interferon response (innate immunity) ensures a protection against the virus and limit the virus spreading from the gastrointestinal tract to the CNS (177, 178). Moreover, the adaptive immunity stimulated by the vaccine was found to trigger plasmocytes able to secrete neutralizing antibodies which specifically recognize epitopes or “sites” on the capsid proteins of polioviruses (site 1 is a linear epitope in VP1; sites 2 and 3 are discontinuous epitopes formed from loops contributed by different capsid proteins), also known as major “neutralizing antigenic sites” (N-Ags) (179–181), IPV and OPV induce similar responses against site 1, while OPV induces a better response than IPV for site 3 and longer B-cell memory (182–185). Neutralizing antibodies were reported to bind both WPV1 and WPV2 and WPV3 with lower affinity (186). The induction of mucosal enteric neutralizing IgA is crucial to limit the magnitude and duration of virus shedding (187, 188). A T cell response to the antigenic structure in VP1 and other capsid proteins have been reported (189, 190). In the USA, the risk of VAPP was estimated at one case per 2.4 million OPVs doses administered (191). In contrast, the risk of VAPP is about 100- to 3,000-fold increased in patients with immunodeficiency (192, 193). While the period of virus shedding is usually 2 to 6 weeks in immunocompetent individuals, virus excretion can be prolonged for years in immunocompromised individuals (137, 194). Moreover, genetic divergence can be associated with changes in the antigenic surface of the viruses, which can lead to resistance to neutralization by the immune system (195, 196). Robustness of WPV1 against vaccine-derived antibodies has been well documented for a large outbreak of poliomyelitis with unusual 47% lethality that occurred in Pointe Noire, Republic of Congo in 2010 (197).

After decades of the global application of a massive anti-polio immunization campaigns (using tOPV followed by the “switch” to bOPV), poliomyelitis due to WPV is much less frequent worldwide with only rare geographical exceptions. However, over time it was clearly established that OPVs can circulate in under-vaccinated communities with a risk that it may genetically revert to a neuropathogenic phenotype. A recent paper investigating 15,331 stool samples from children receiving OPV and their contacts reported that 61% of OPV1, 71% OPV2, and 96% OPV-3 samples with available data (122 samples) had evidence of a reversion at the key 5′ UTR attenuating position and 28% of OPV1, 12% OPV2, and 91% of OPV3 wth available data (337 samples) had ≥1 known reversion mutations in the VP1 gene (198). Considering the risk-benefit ratio, for several decades the reversion of the OPVs was an unavoidable price to pay for obtaining an effective poliovirus vaccine linked to major success in controlling disease, despite a highly mutable RNA genome of a virus with human gut tropism. So long as one person is infected by a poliovirus there is a risk that polio can reappear, even in places where it had already been eradicated. The circulation of vaccine-derived polioviruses can be rapidly stopped with two or three rounds of OPV immunization campaigns. The risk of the reintroduction of poliovirus through interspecies circulation of the virus should not be underestimated, even if it seems very low due to the fact that the host range of polioviruses is considered to be restricted to humans and simians as a consequence of receptor polymorphism in mammals (160). An outbreak encirclement control strategy would imply that it must be combined with syndromic surveillance systems and flawless monitoring of poliovirus in wastewater by routine screening from sewers and extensive whole genome sequencing of isolates. This is a crucial element for a very early warning of a risk of outbreak.

Already in the 1990s, the use of OPVs in regions with rare cases of poliomyelitis was beginning to be disputed and the demand to produce safer vaccine strains of improved genetic stability was considered a serious issue (58). Ninety per cent of cVDPV revertants isolated belonged to OPV2 and OPV3 types. In 2014, Yan et al. (96) reached the conclusion that due to the risk of cVDPV2, the OPV2 should be removed from the trivalent OPVs formulation. Attempts have been made to seek solutions to the problem, including the cessation of OPV2 vaccination through removing the OPV2 from the tOPVs formulation and its replacement by IPV, which is safer but induces lower level of mucosal immunity specifically in the gut where the polioviruses reside. Clinicians have had a long history of IPV use since the formalin IPVs was licensed in 1955 for use in the United States, Canada, and Western Europe although its use had declined in the US after the introduction of tOPVs. However, its use was maintained in some countries in Northern Europe (Finland, Iceland, Sweden, and the Netherlands) and Canada. In 1997, faced with the problem of cVDPV, the USA shifted from tOPVs to a sequential IPV/OPV schedule, replaced 3 years later by an all-IPV schedule (199). Manufacturers from different countries have developed IPV1, IPV2, and IPV3, including Glaxo Smith Kline, Biological SA, USA (trade name: Poliorix), Sanofi Pasteur SA, France (trade name: IMOVAX POLIO), Serum Institute of India Pvt. Ltd., India, and Bilthoven Biologicals B.V., The Netherlands, among others (200). The inactivated vaccines (known to be more than five times more expensive than the oral live attenuated vaccine) can be administered by injection into the arm or leg.

However, it is also considered that only high-income countries with a limited risk for fecal-oral WPV transmission can maintain high enough population immunity to transmission using IPV in their routine program (201). Indeed, IPV offers good protection against paralysis but is much less effective than the oral vaccine to trigger mucosal immunity and stop virus circulation, potentially allowing silent virus circulation (endemicity of the virus) without detection of paralytic cases for long periods of time. With this transition in mind, a variety of models have looked to explore the risks associated with the circulation of OPVs and the probability of emergence of cVDPVs (202–205). The Duintjer Tebbens–Thompson model program (201) suggested that if iVDPV or other live poliovirus reintroductions occurred long after the cessation of OPV and/or in places with conditions conducive to intense fecal-oral poliovirus transmission, based on 1,000 stochastic iterations and a somewhat arbitrary threshold of 50,000 post-cessation polio cases, then this would result in uncontrolled outbreaks and a need to restart tOPVs globally. Notably, Israel was declared a “polio-free” country by the WHO and had to take stringent surveillance measures when faced a polio outbreak in 2013–2014 in Rahat, the largest predominantly Bedouin city, when a WPV1 was introduced from Pakistan in late 2012 (206). Israel launched a supplementary vaccination campaign with bOPV at the beginning of August 2013, and the outbreak ended in early 2014. From 2016 to 2019 withdrawal of OPV2, 377 cVDPV2 cases of AFP were reported across 17 countries of which nine were reported to be free of type 2 poliovirus circulation between 2000 and 2016 (207). A recent study based on environmental surveillance of type 2 poliovirus in London sewage between February and July 2022, isolated 118 genetically linked cVDPV2 samples (a recombinant genetic structure between cVDPV2 with reversion of the two main OPV2 attenuation mutations and an unidentified species C enterovirus with a crossover point at nucleotide 5,139) in multiple sites from north and east London, suggesting that such strains are likely to have reversed to neurovirulence and are potentially able to cause paralytic poliomyelitis (142). Other similar events were recorded in 2022 in Israel and the USA, where the first paralytic poliomyelitis cases since 1989 and 2013, respectively, have been reported (208).

Regarding the OPV3, it converts the strong C472:G537 pair in RNA secondary structure to a weak U472:G537 pair. The rapid back-mutation at nucleotide 472 in the 5′-NCR of OPV3 (selection pressure against the U at this position within the RNA secondary structure), suggests that the OPV3 strain is debilitated from growing in intestinal cells. It was proposed to replace the C472:G537 by U472A537 to weaken the stem and have an attenuating effect (58). Serial passages of poliovirus in the presence of the base analog ribavirin were reported to lead to the selection of a polymerase variant (G64S) with threefold increased 3D^pol^ fidelity (209). An improved OPV3 formulation was developed by Pfizer Laboratories in the UK after extracting the RNA from low-passage OPV3, transfecting monkey kidney cells, and screening plaques for low neurovirulence. The new seed stock was considered to be more stable during production and was also free of SV40 contamination (73). Thus, Vignuzzi et al. (210) proposed to engineer attenuated virus vaccines with increased replication fidelity to improve their safety. Notably, it was reported that a G64S mutation in enterovirus ENV-71 also confers increased RNA polymerase fidelity (211).

Another amino acid replacements such as L123F in ENV-71 was also shown to modify the replication fidelity of this virus (212). Replication of coxsackievirus CBV-B3 in the presence of ribavirin or 5-azacytidine also selected a fidelity variant in the viral polymerase (A372V) (213). More recently, it was suggested to develop the strategy of a “codon-deoptimized new OPV2 candidate” (nOPV2-CD) to further attenuate OPV2 by changing preferred codons across the capsid to non-preferred synonymous codons (143). Before the cessation of OPV2 vaccination, vaccine-associated paralytic polio was estimated to cause between two and four cases per million live births per year in countries vaccinating with OPVs (214). Currently, WPV1 is believed to be the only serotype still in circulation and questions can be raised as to whether the strategy implemented to achieve immunization against WPV remains appropriate. The planned strategy of replacing the OPV formulation with the nOPV formulation partially addresses the problem but does not totally eliminate the risk of reversion by recombination. Advances in genetics and biotechnology have made considerable progress and could be considered to identify potential new targets for novel anti-enteroviral drugs and to design new safe and effective vaccine formulations based on: (i) viral vector vaccines such as adenovirus (a virus with intestinal cell tropism) expressing the recombinant capsid protein or capsid neutralizing epitopes of polioviruses or Newcastle disease virus-based vaccine; (ii) protein-based vaccines (such as recombinant capsid proteins or epitope-based synthetic polypeptides), which are delivered by polymers or liposomes microparticles; (iii) RNA vaccine (RNA coding for viral structural proteins) or (iv) bacteriophage-based vaccines (215–219). Each method has its advantages and disadvantages, and in each case the benefit/risk ratio must be assessed. It is crucial that the international health authorities encourage manufacturers to produce new types of vaccines inducing good mucosal immunity to definitively eradicate poliomyelitis while avoiding the use of live polioviruses that can revert by recombination.

## 15 Conclusion

The development of a live attenuated poliovirus vaccine to fight poliomyelitis was a great step forward, and has produced spectacular results with the eradication of poliomyelitis from most countries. For almost half a century, this was the best strategy for stopping the circulation of WPVs, following the successful example of smallpox eradication. WPV2 has been globally eradicated and the last report of WPV3 date from November 2012. The WPV1 remains the only circulating wild strain. Over the past decade, the global spread of poliomyelitis has evolved to the point that severe polio cases reported today are more frequently linked to man-made attenuated OPV vaccination and the emergence of cVDPVs (e.g., 232 cases in 2018; 375 in 2019; 1,117 in 2020; 680 cases in 2021; 666 cases in 2022) than to WPVs (e.g., 33 cases in 2018; 176 cases in 2019; 140 cases in 2020; five cases in 2021; 30 cases in 2022). Moreover, genetic studies of circulating cVDPVs have highlighted frequent recombination between cVDPVs and other enteroviruses sharing the same ecological niche, thereby increasing the genetic diversity of circulating viruses.

We are now at a critical moment in the polio endgame with a problem of vaccine coverage and emergence of revertants. Immunity against polioviruses is not yet universal and many under vaccinated countries are exposed to a vulnerability to polioviruses and a risk of pandemic. Many children never receive basic vaccination because unreachable by vaccination teams and circulating poliovirus strains in under vaccinated populations can serve as a reservoir that can cause outbreaks in sub-optimally immunized populations. Faced with the reality of frequent mutation and recombination events, it may be appropriated to rethink the polio vaccination strategy, and question whether the use of OPVs or even relatively better attenuated OPVs (e.g., nOPVs), remains reasonable. When a vaccine dose contains several tens of thousands of viruses and hundreds of millions of doses are injected, the risk of facilitating the emergence of a pathogenic virus is not negligible (a risk estimated at one case per 2.4 million OPVs doses administered). Poliomyelitis cases induced by vaccination are no longer acceptable today. The current plan of the WHO Strategic Advisory Group of Experts is to withdraw bivalent OPV within 3 years after the circulation of WPV1 is stopped, and then continue immunizations with IPVs only (more than five times more expensive than the OPVs) for 10 years after withdrawal of OPVs. While waiting for new vaccines, it might be desirable to seek to control outbreaks rapidly based on the encirclement method (as is the case with the EUL programme) rather than continuing to promote large-scale injection campaigns of live attenuated viruses, leading to this paradox that vaccination induces more cases of polio than the indigenous WPVs. This is the simple application to polio of the One Health/EcoHealth concept. However, this strategy still clashes with reality in resource-limited countries that are relying on OPV vaccination to prevent the endemic transmission of live polioviruses.

## Author contributions

CD: Conceptualization, Writing – original draft. PP: Writing – review & editing. AL: Writing – review & editing. PC: Writing – review & editing. DR: Conceptualization, Writing – original draft.
